# Clinical, Microbiological, and Outcome Characteristics of Pediatric Patients With Candidemia at the Pediatric Hospital of the National Medical Center Siglo XXI From 2022 to 2025

**DOI:** 10.7759/cureus.108978

**Published:** 2026-05-16

**Authors:** Grace A Montes García, Daniel Pacheco Rosas, Raúl Romero Feregrino

**Affiliations:** 1 Department of Infectious Diseases, Instituto Mexicano del Seguro Social, Mexico City, MEX; 2 Department of Pediatrics/Infectious Diseases Service, Centro Médico Nacional Siglo XXI, Mexico City, MEX; 3 Department of Pediatrics, Mexican Academy of Pediatrics, Mexico City, MEX

**Keywords:** antifungal therapy, candida species, candidemia, healthcare-associated infections, pediatric infection

## Abstract

Introduction: Candidemia is a major healthcare-associated infection in pediatric populations, driven by invasive medical interventions and shifts in fungal epidemiology toward non-albicans Candida species. Mortality remains high worldwide, particularly in infants and critically ill children. Updated regional data from tertiary-care centers are essential to guide prevention and treatment strategies.

Methods: An observational, retrospective, cross-sectional study was conducted at the Pediatric Hospital of the National Medical Center Siglo XXI (2022-2025). Patients aged 0-17 years with at least one positive blood culture for Candida spp. were included. Exclusion criteria comprised bacterial coinfection, palliative care, absence of antifungal treatment, incomplete clinical records, and diagnoses made at external institutions. Clinical characteristics, risk factors, species distribution, antifungal susceptibility, treatment, and outcomes were analyzed.

Results: A total of 99 patients were evaluated, of whom 42 met the inclusion criteria. Of these, 28 (66.7%) were male patients, and the most frequent age group was infants, with 20 patients. The main risk factors were central venous catheter use in 42 patients (100%), prior antibiotic use in 39 patients (92.9%), mechanical ventilation in 35 patients (83.3%), and ICU stay in 33 patients (73.8%). Non-albicans species predominated with 34 isolates (80.9%), with *Candida tropicalis* being the most frequent, 14 isolates (33.3%). Resistance to fluconazole was low, one isolate (2.4%). The most commonly used treatment was fluconazole, administered to 24 patients (57.1%). In-hospital mortality was 11 patients (28.6%).

Conclusion: Candidemia in this pediatric cohort was predominantly caused by non-albicans Candida species, particularly *C. tropicalis*. Risk factors remained strongly linked to invasive medical support and prior antibiotic exposure. Despite appropriate management, mortality remained substantial, emphasizing the need for early recognition, optimized antifungal selection, and strengthened diagnostic and susceptibility testing practices in tertiary pediatric care settings.

## Introduction

Candidemia is a significant healthcare-associated infection caused by opportunistic yeasts of the Candida genus, which normally colonize human mucosa and skin but may invade the bloodstream under specific host conditions [1-6]. In the United States, approximately 25,000 cases occur annually, with an incidence of nearly nine per 100,000 inhabitants and hospital mortality around 25% [7]. Pediatric and neonatal populations experience substantial disease burden, with incidences up to 5.1 per 10,000 admissions and mortality exceeding 25% [8-11].

Risk factors are closely tied to medical care, including central venous catheters, broad-spectrum antibiotics, mechanical ventilation, parenteral nutrition, and immunosuppression [12,13]. The epidemiology has shifted from predominance of *Candida albicans* to increasing non-albicans species such as *Candida tropicalis*, *Candida​​​​​​​ parapsilosis*, and *Candida​​​​​​​ glabrata*, which differ in virulence and antifungal susceptibility [14-17]. Blood cultures remain the diagnostic gold standard [18,19], and treatment is guided by Infectious Diseases Society of America recommendations, with echinocandins as first-line therapy [1,18-21].

Studies from Australia, Turkey, Chile, and Mexico demonstrate wide variability in species distribution, susceptibility, and mortality [14,22-24], underscoring the need for updated regional data, particularly in pediatric tertiary-care settings. The aim is to describe the clinical, microbiological, therapeutic characteristics and outcomes of pediatric patients with candidemia at the Pediatric Hospital of the National Medical Center Siglo XXI during the period from 2022 to 2025.

## Materials and methods

An observational, retrospective, cross-sectional study was conducted. Children aged 0-17 years, 11 months, and 29 days with peripheral or central blood cultures positive for Candida were included. The primary objective of this study was to characterize the clinical presentation, microbiological profile, antifungal management, and outcomes of pediatric patients with candidemia at the Pediatric Hospital, Centro Médico Nacional (CMN) Siglo XXI, between 2022 and 2025. Secondary objectives were to describe the distribution of Candida species isolated from pediatric blood cultures, identify the most relevant risk factors associated with candidemia, evaluate antifungal treatment patterns and azole resistance rates, and estimate in-hospital mortality.

Patients with bacterial coinfection at the time of candidemia, those with Candida-positive blood cultures who did not receive antifungal treatment, and patients who were discharged before completing diagnostic workup and/or treatment.

Patients were identified through a database obtained from the Pediatric Infectious Diseases Service covering the period from January 2022 to August 2025. Medical records were reviewed to identify patients who met the inclusion criteria. The hospital’s pediatric clinical archives were accessed to review and extract information from the records of eligible cases.

Data were recorded in a data collection form specifically designed for the study. Once complete, the data were entered into an electronic database, and statistical analysis was performed. Descriptive statistics were used for quantitative variables (means, medians, and standard deviation) and qualitative variables (frequencies and percentages). Database construction and analysis were conducted using Statistical Package for the Social Sciences version 29 (IBM Corp., Armonk, NY).

## Results

**Figure 1:**
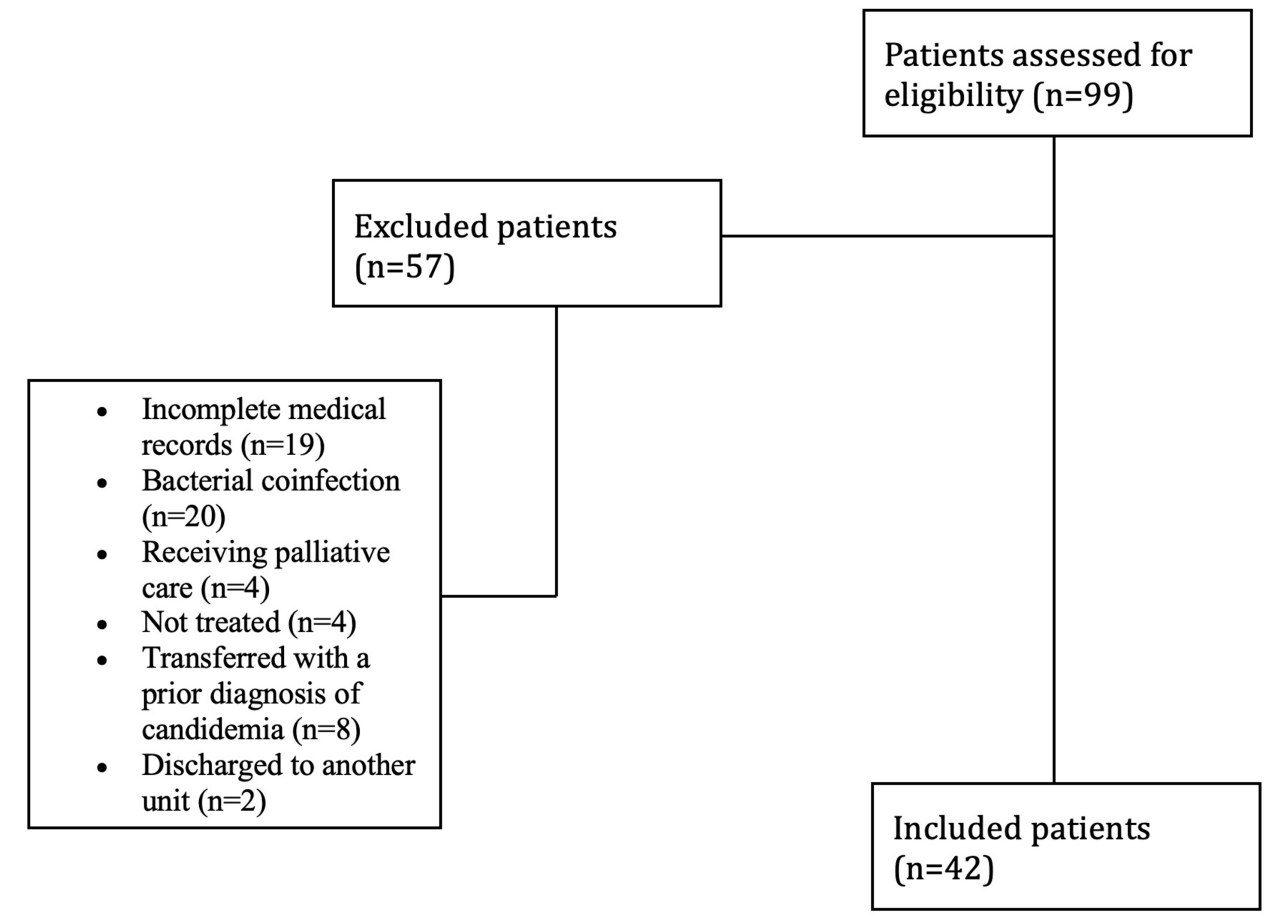
Selection process

Between January 2022 and August 2025, 99 patients with positive blood cultures for Candida spp. were identified, of whom 42 met the inclusion criteria. Exclusions included bacterial coinfection (n = 20), prior diagnosis from another institution (n = 8), palliative care (n = 4), absence of antifungal treatment (n = 4), discharge to another unit without follow-up (n = 2), and incomplete records (n = 19). The selection process is shown in Figure 1.

Most were male patients (n = 28, 66.7%). Infants represented the largest age group (45.2%, n = 19), followed by school-aged children and adolescents (19.0% each, n = 8), and preschool-aged children (16.7%, n = 7). Nutritional status at admission was predominantly normal (45.2%, n = 19), with severe malnutrition in 38.1% (n = 16). Table 1 summarizes baseline characteristics.

**Table 1:** Clinical and demographic characteristics of the study population

Characteristic	n (%)
Male sex	28 (66.7)
Female sex	14 (33.3)
Infants	19 (45.2)
Preschool children	7 (16.7)
School-age children	8 (19.0)
Adolescents	8 (19.0)

Central venous catheter use was present in 97.6% (n = 41), with colonization documented in 85.3% (n = 35); the median duration prior to candidemia was 21 days (range, 6-56). Prior exposure to broad-spectrum antibiotics occurred in 92.9% (n = 39; median 14 days, range 3-67). Invasive mechanical ventilation was reported in 83.3% (n = 35; median 12 days, range 1-120), and ICU stay in 73.8% (n = 31; median 13 days, range 2-164). Additional exposure durations are shown in Table 2.

**Table 2:** Clinical exposure durations

Characteristic	Median (min-máx)
Days of central venous catheter use	21 (6-56)
Days of antibiotic use	14 (3-67)
Days of mechanical ventilation	12 (1-120)
Days of ICU stay	13 (2-164)
Days of parenteral nutrition use	20 (5-150)
Days of immunosuppressive therapy	5 (5-39)
Days of neutropenia	13 (7-20)

At diagnosis, fever was the most frequent clinical manifestation (83.3%, n = 35), followed by tachycardia (76.2%, n = 32), thrombocytopenia (33.3%, n = 14), leukocytosis (23.8%, n = 10), and tachypnea (21.4%, n = 9). Clinical manifestations are shown in Table 3.

**Table 3:** Clinical manifestations at diagnosis in pediatric patients with candidemia

Manifestations	n (%)
Fever	35 (83.3)
Tachycardia	32 (76.2)
Thrombocytopenia	14 (33.3)
Leukocytosis	10 (23.8)
Tachypnea	9 (21.4)
Skin lesions	2 (4.8)
Altered mental status	2 (4.8)
Hypothermia	1 (2.4)

*C. tropicalis* was the most commonly isolated species (33.3%, n = 14). *C. albicans* and *Candida pelliculosa *were each identified in 19.0% (n = 8). *C. parapsilosis* and *Candida lusitaniae* accounted for 11.9% each (n = 5), and *C. glabrata* was isolated in one case (2.4%). Fluconazole resistance was documented in one isolate (2.4%), while susceptibility data were not available in 38.1% (n = 16). Species distribution is shown in Table 4.

**Table 4:** Distribution of Candida species in pediatric candidemia isolates

Variable	n (%)
Candida species	
Candida tropicalis	14 (33.3)
Candida albicans	8 (19.0)
Candida pelliculosa	8 (19.0)
Candida parapsilosis	6 (14.3)
Candida lusitaniae	4 (9.5)
Candida glabrata	1 (2.4)
Candida krusei	1 (2.4)
Total non-albicans Candida isolates	34 (80.9)
Total *Candida albicans* isolates	8 (19.0)

Fluconazole was the most frequently administered antifungal (57.1%), followed by caspofungin (23.8%) and amphotericin B (11.9%). Treatment duration ranged from 5 to 28 days, commonly between 14 and 20 days. Median time to blood culture clearance was six days (range 5-11).

Overall, 32 patients (76.2%) achieved clinical cure, defined as resolution of clinical signs and symptoms of infection (e.g., fever or hemodynamic instability), along with documented clearance of candidemia, as evidenced by negative follow-up blood cultures after initiation of antifungal therapy and no need for escalation of antifungal treatment. Twelve patients (28.6%) died during hospitalization; 11 deaths (26.2%) occurred within 30 days of candidemia onset, and five deaths (11.9%) were directly attributable to candidemia.

## Discussion

In this cohort of 42 pediatric patients with candidemia, the infection remained strongly associated with healthcare exposure, consistent with international findings. Infants represented the most affected age group, accounting for nearly half of all cases. This pattern aligns with historical data from the same institution, where infants were also the predominant group, and with global reports in which distribution varies by region but often affects neonates, infants, and adolescents [14,23]. Although the present study showed a predominance of male patients, no consistent sex distribution has been demonstrated in previous series [13,14,24].

The most frequent associated factors were central venous catheter use and previous exposure to broad-spectrum antibiotics, both of which occurred in more than 90% of cases. These rates are comparable to those reported in high-income and middle-income countries and underscore the role of invasive devices and antimicrobial pressure in the development of candidemia [8,22]. The proportion of patients with central venous catheters was higher than that reported in this hospital two decades ago [14], suggesting an increasing reliance on invasive support in critically ill children. Invasive mechanical ventilation was also common, consistent with Rajeshwari et al. [24], who identified it as a major risk factor in pediatric intensive care settings.

Clinical presentation was dominated by fever and tachycardia, followed by thrombocytopenia, leukocytosis, and tachypnea. These findings reflect the nonspecific nature of invasive Candida infections, which require a high index of suspicion based on risk factors and supportive microbiological evidence [7,18]. A subset of patients exhibited localized organ involvement, including hepatosplenic, ocular, cutaneous, and cardiac disease, consistent with complicated candidemia. Current guidelines recommend systematic evaluation for metastatic complications because these findings influence treatment duration and prognosis [7,18].

Non-albicans Candida species predominated, representing more than 80% of isolates. *C. tropicalis* was the most frequent species, followed by *C. albicans* and *C. pelliculosa*. Fluconazole resistance was low; however, susceptibility testing was unavailable in over one-third of isolates, limiting interpretation. The shift toward non-albicans species aligns with global epidemiologic trends and reinforces the need for reliable susceptibility testing to guide therapy [14,19].

Fluconazole was the most frequently used antifungal, followed by caspofungin and amphotericin B. Although fluconazole remains widely used in resource-limited settings [23], the high proportion of non-albicans species and the lack of susceptibility data support reconsidering echinocandins as first-line therapy, consistent with international guidelines [24]. Treatment duration typically fell within recommended ranges.

Despite appropriate management, 30-day mortality (26.2%) exceeded that reported in large pediatric cohorts from high-income countries (~12%) [22]. Mortality in this series more closely resembles outcomes in hematologic or high-risk pediatric populations [3]. Nevertheless, more than three-quarters of patients achieved a cure, highlighting the importance of early diagnosis, appropriate antifungal therapy, and timely removal of invasive devices. This study provides updated local data and underscores the need for improved diagnostic practices, routine susceptibility testing, and prospective surveillance.

Among the strengths of this study are that it represents the most recent series of pediatric candidemia at the Hospital de Pediatría del CMN Siglo XXI, updating information generated nearly two decades ago, and that it integrates clinical, microbiological, therapeutic, and outcome data simultaneously, providing a comprehensive view of the problem. Additionally, it was conducted in a national referral center, making the findings relevant for other tertiary care units in Mexico.

Among the limitations, the retrospective design should be considered, as it depends on the quality and completeness of medical records and may result in missing information (e.g., antifungal susceptibility testing and incomplete clinical records in the archives due to the high patient demand in our unit). Furthermore, the relatively small sample size (n = 42) limits the statistical power to detect robust associations between specific risk factors and mortality. Finally, there was a lack of complete data on some potential prognostic factors, such as time to initiation of antifungal therapy from blood culture collection, systematic catheter removal, and baseline functional status, among others.

## Conclusions

Non-albicans Candida species were the most frequent cause of candidemia in this cohort, with *C. tropicalis* identified as the predominant species. The main factors associated with candidemia were consistent with previous reports, with central venous catheter use as the leading risk factor, followed by prior exposure to broad-spectrum antibiotics and invasive mechanical ventilation. Fluconazole was the most commonly administered antifungal agent, used in more than half of patients; documented resistance was low (2.4%), although susceptibility data were unavailable in 38.1% of isolates. Candidemia-related mortality was 11.9%, which is lower than rates reported two decades ago in the same institution but remains higher than those described in high-income countries.
